# Simulation of the effect of mucociliary clearance on the bronchial distribution of inhaled radon progenies and related cellular damage using a new deposition and clearance model for the lung

**DOI:** 10.1007/s00411-020-00868-5

**Published:** 2020-08-31

**Authors:** Árpád Farkas

**Affiliations:** Environmental Physics Department, Centre for Energy Research, Konkoly-Thege M. út 29-33, 1121 Budapest, Hungary

**Keywords:** Radon inhalation, Fast clearance, Cell death, Cell transformation

## Abstract

Most of the current dosimetry models of inhaled short-lived radon decay products assume uniform activity distributions along the bronchial airways. In reality, however, both deposition and clearance patterns of inhaled radon progenies are highly inhomogeneous. Consequently, a new deposition-clearance model has been developed that accounts for such inhomogeneities and applied together with biophysical models of cell death and cell transformation. The scope of this study was to apply this model which is based on computational fluid and particle dynamics methods, in an effort to reveal the effect of mucociliary clearance on the bronchial distribution of deposited radon progenies. Furthermore, the influence of mucociliary clearance on the spatial distribution of biological damage due to alpha-decay of the deposited radon progenies was also studied. The results obtained demonstrate that both deposition and clearance of inhaled radon progenies are highly non-uniform within a human airway bifurcation unit. Due to the topology of the carinal ridge, a slow clearance zone emerged in this region, which is the location where most of the radio-aerosols deposit. In spite of the slow mucus movement in this zone, the initial degree of inhomogeneity of the activity due to the nonuniform deposition decreased by a factor of about 3 by considering the effect of mucociliary clearance. In the peak of the airway bifurcation, the computed cell death and cell transformation probabilities were lower when considering deposition and clearance simultaneously, compared to the case when only deposition was considered. However, cellular damage remained clustered.

## Introduction

Even if the macroscopic exposure level of an individual (e.g. due to radon in air) is known (ICRP 103 2007), the resulting dose distribution within the human body is difficult to quantify because technical and ethical barriers hamper its measurement. In contrast, numerical modelling can be an effective method to quantify the radiation dose at various levels of biological organization (whole body, organs, tissues, cells, subcellular entities). This kind of approach may be feasible especially when the distribution of the energy deposition due to ionising radiation is uneven, like in the case of alpha-particles originating from the decay of inhaled radon and radon progenies. In such a case, the spatial distribution of the induced biological damage may also be non-uniform. Earlier simulations confirmed indeed that, due to the special features of airway morphology and particle aerodynamics, the deposition distribution of inhaled radio-aerosols within the human bronchial airways is highly inhomogeneous (Farkas et al. 2011). Specifically, the number of deposited particles per unit surface (deposition density) can be two or three orders of magnitude higher than the average deposition density, depending on particle size and breathing parameters, among others. In another study (Farkas and Szőke 2013) using inactive insoluble particles, it was demonstrated that the primary deposition patterns of such particles in the lung can be significantly modified by mucociliary clearance. This fast clearance mechanism includes the transport of particles deposited in the lung towards the larynx/pharynx by a thin (a few micrometers thick) mucus layer. This highly viscous mucus layer lines the large bronchial airways and it is propelled by co-ordinated beating of cilia. If the physical half-life of any deposited radioisotope is long enough that it is removed from their initial location of deposition but shorter than the time needed for the mucus to remove them from the airways, then the resulting activity distribution in the airways is different from the primary activity distribution due to deposition alone. In such situations, besides modelling the deposition of radio-aerosols, it is also important to simulate their clearance. Based on current knowledge, short-lived radon progenies are transported by the mucus upward a few centimetres which means that the location of their decay will be different from the location of their deposition (Sturm and Hofmann 2007). In this context it is an important question to what extent mucociliary clearance can modify the highly non-uniform primary activity distribution due to deposition. In addition, it is important to clarify the role of mucociliary clearance in the cellular processes following the inhalation and decay of radon progenies. To reveal any effects of mucociliary clearance, a complex deposition-clearance-decay model has been developed and applied in the present study, allowing for a continuous activity size distribution of radon progeny, and for a dynamic, i.e., time-dependent, lung deposition and clearance. Specifically, the current standard model was refined in that it accounts for a realistic measured size distribution of radon progenies instead of assuming a simplified bimodal distribution, similar to the approach followed by (James et al. 2004).

## Methods

### Airway and mucus geometry

**Fig. 1:**
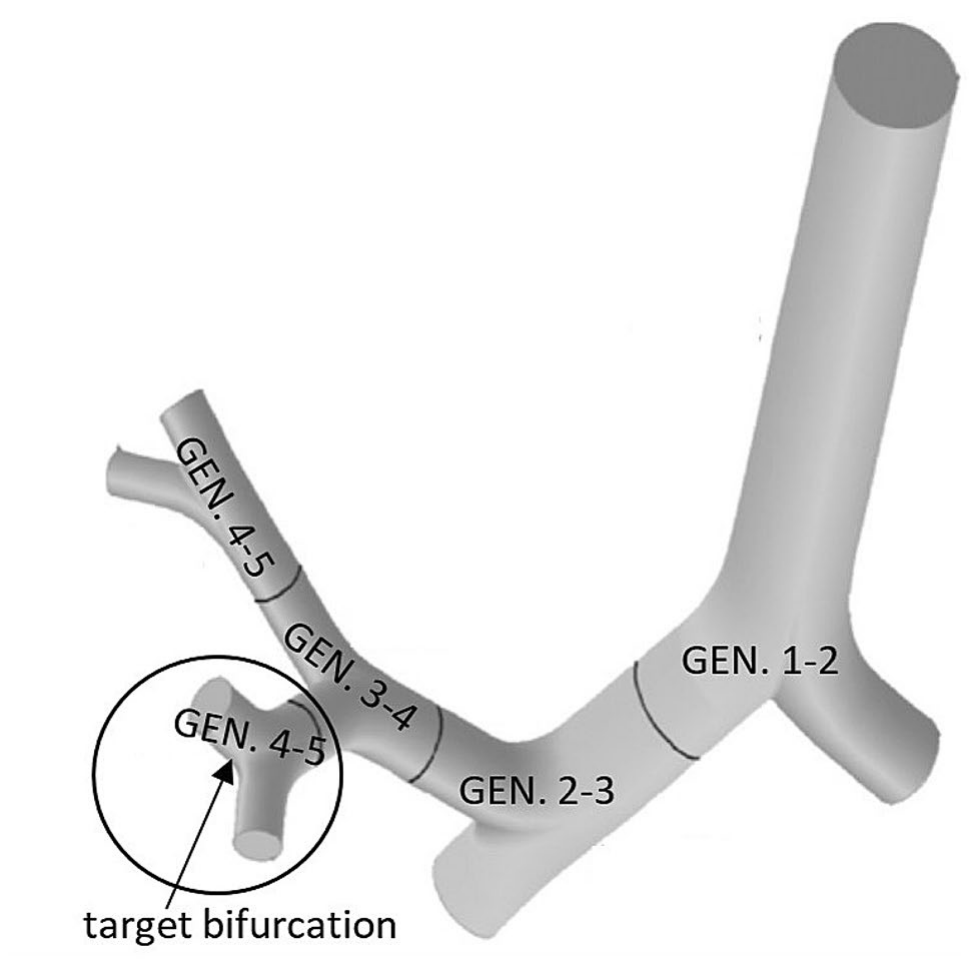
Location of the modelled airway bifurcation within the tracheobronchial tree

To study the effects associated with deposition, clearance and decay of inhaled short-lived radon progenies, a representative bronchial airway bifurcation has been used. The morphometric data of this symmetric airway segment corresponding to the 4th and 5th airway generations (trachea is the first generation) are summarized in Table 1. The method used to construct this geometry is described in detail in Hegedűs et al. (2004). As depicted in Fig. 1, the modelled single airway bifurcation is located in the right upper lobe of the lung. Its parent branch is a segmental bronchus, while its daughter branches are sub-segmental bronchi (anterior and posterior). This central airway geometry has been chosen because the maximum of the deposition density is in this region of the lungs (Füri et al. 2020) and according to earlier histopathological studies (Veeze 1968; Garland 1962) malignancies due to radon inhalation were most frequent in this area. Though the modelled airway bifurcation is only a small part of the central airways, similar tendencies are expected to occur in all the other bifurcation units of the lung. The modelled bifurcation will be referred to as “target bifurcation” or “model bifurcation” throughout the manuscript.

**Table 1:** Geometric data of the model airway bifurcation

Length of parent branch (cm)	Length of daughter branch (cm)	Diameter of parent branch (cm)	Diameter of daughter branch (cm)	Bifurcation angle (°)	Curvature radius of daughter branch (cm)	Carinal curvature radius (cm)
0.72	1.20	0.56	0.45	35.0	1.43	0.1

The modelled airway bifurcation was covered by a mucus layer. Actually, this layer is composed of two sublayers, namely the preciliary layer (sol layer) and the gel layer that resides atop the sol layer. The composition and movement of the two layers is different (Craster and Matar 2000; Button et al. 2012). In the present work, only the movement of the gel layer was modelled, since this layer transports any radioactive particles. The thickness of the gel layer was assumed to be 5 µm. Although the movement of the sol layer was not modelled, this layer was considered when the interaction of particles with the epithelium was simulated, because this layer can absorb a part of the energy of emitted alpha-particles. The thickness of the sol layer was assumed to be 4 µm.

### Whole airway deposition and clearance modelling

It should be noted that, although the distribution of particles and the related biological endpoints were modelled in a single airway bifurcation, a part of the particles originated from deeper airways. To simulate the realistic number and type of particles entering the model bifurcation per unit time for different exposition scenarios, deposition and clearance of these particles must be known at the level of the whole lung.

For the modelling of the number of particles deposited in the upper airways and in each airway generation, the Stochastic Lung Model was used. This model requires as main input the lung capacity and some breathing parameters, and parameters characterizing the aerodynamics of the inhaled particles. The output of the model includes the fractions of particles deposited in different anatomical regions of the lung. In addition, the model provides the deposition fractions specific to each airway generation. In the model the lengths, diameters and branching angles of the airways are selected by Monte Carlo techniques from statistical distributions established based on morphometric data measured at the Lovelace Inhalation Toxicology Research Institute (Raabe et al. 1976). The evaluation of these data is described in Koblinger and Hofmann (1985). In the same publication, criteria for the simulation of particle pathways are described (typically, trajectories of 100,000 particles should be simulated). It is worth noting that in the model the particles are tracked only in the bronchial and acinar airways, but not in the extrathoracic airways. Instead, the model applies empirical deposition formulae, to quantify the particle deposition in the upper airways. In the present work, Cheng’s diffusional deposition formula was used (Cheng 2003) in conjunction with Yu’s deposition formula (Yu et al. 1981) providing the deposition as a function of the impaction parameter. In the bronchial and acinar regions, particles deposit by inertial impaction, gravitational sedimentation and Brownian diffusion. Due to their small size, unattached radon progenies deposit by thermal diffusion. Radon progenies attached to the environmental particles deposit by all the three mechanisms, though deposition by diffusion is predominant. The equations describing the deposition by these three mechanisms are provided in Koblinger and Hofmann (1990), and were adopted from the work of Yeh and Schum (Yeh and Schum 1980).

Clearance of particles deposited in the acinar airways was not considered in the present study. Clearance of particles deposited in the tubular airways was modelled by a simple algorithm which considered the computed airway generational deposition fractions, the length of the airway tube, the velocity of the mucus in the corresponding airway generations (Hofmann and Sturm 2004), and the activity of the deposited radon progenies. Denoting the number of ^218^Po, ^214^Pb and ^214^Bi/^214^Po isotopes at the beginning of simulations by $N_{1}^{0}$, $N_{2}^{0}$ and $N_{3}^{0}$, respectively, the number of these isotopes after time *t* can be calculated by Eqs. 1–3:

1 $$N_{1} (t) = N_{1}^{0} e^{{ - \lambda_{1} t}} ,$$

2 $$N_{2} (t) = \frac{{\lambda_{1} }}{{\lambda_{2} - \lambda_{1} }}N_{1}^{0} (e^{{ - \lambda_{1} t}} - e^{{ - \lambda_{2} t}} ) + N_{{_{2} }}^{0} e^{{ - \lambda_{2} t}}$$

and

3 $$N_{3} (t) = \frac{{\lambda_{1} \lambda_{2} }}{{\lambda_{2} - \lambda_{1} }}N_{1}^{0} \left( {\frac{{e^{{ - \lambda_{1} t}} - e^{{ - \lambda_{3} t}} }}{{\lambda_{3} - \lambda_{1} }} - \frac{{e^{{ - \lambda_{2} t}} - e^{{ - \lambda_{3} t}} }}{{\lambda_{3} - \lambda_{2} }}} \right) + \frac{{\lambda_{2} }}{{\lambda_{3} - \lambda_{2} }}N_{{_{2} }}^{0} \left( {e^{{ - \lambda_{2} t}} - e^{{ - \lambda_{3} t}} } \right) + N_{3}^{0} e^{{ - \lambda_{3} t}} ,$$

where $\lambda_{1}$, $\lambda_{2}$ and $\lambda_{3}$ are the physical decay constants of the three isotopes. In Eqs. 1–3, *t* represents the time needed for an isotope to reach the model bifurcation after its deposition in one of the airway generations with a generation number higher than 5. Daughter isotopes reaching the end of the decay chain (^210^Pb) were discounted. The number and type of the radon progenies reaching the 5^th^ generation airways per unit time were stored.

### Local deposition and clearance modelling

The deposition of radon progenies in the target bifurcation was simulated by CFPD (computational fluid and particle dynamics) techniques. For this purpose, the ANSYS FLUENT code was used in conjunction with user-defined subroutines (written in C++ language and attached to the original code) to tailor the code to the special needs of the present tasks. Details of this technique are described for example in Farkas and Balásházy 2008. Briefly, the 3D airway geometry was spatially discretized by the application of an unstructured numerical mesh. The inhaled air was treated as a continuum (Euler method), its pressure, velocity and turbulence parameters were computed in each computational cell by solving the equations of mass and momentum conservation and the turbulence equations. The inhaled particles were discrete entities (Lagrange method). There was a one-way coupling between the two phases (air-particle). Particle trajectories were allowed to be influenced by airflow, but the particles had no influence on the airflow.

Clearance of particles within the target bifurcation was modelled also by CFPD techniques using the Euler–Lagrange approach. Meshing of the mucus layer is quite a challenging task due to its low thickness (see Farkas and Szőke 2013). The movement of the mucus layer was modelled assuming a density of 100 kg/m^3^, a viscosity of 1 Pa s (Podgórski and Sosnowski 2000), and a mucus velocity of 1 mm/min (Asgharian et al. 2001) at the entrance of fifth generation branches. This value was derived from a mucus velocity of 5.5 mm/min measured in the trachea (Cuddihy and Yeh 1988). Due to the high viscosity of the mucus, the trajectories of the particles mostly follow the streamlines of the mucus flow field. More details about the CFPD clearance model can be found in Farkas and Szőke (2013).

### Dynamic model of deposition, clearance and decay

**Fig. 2:**
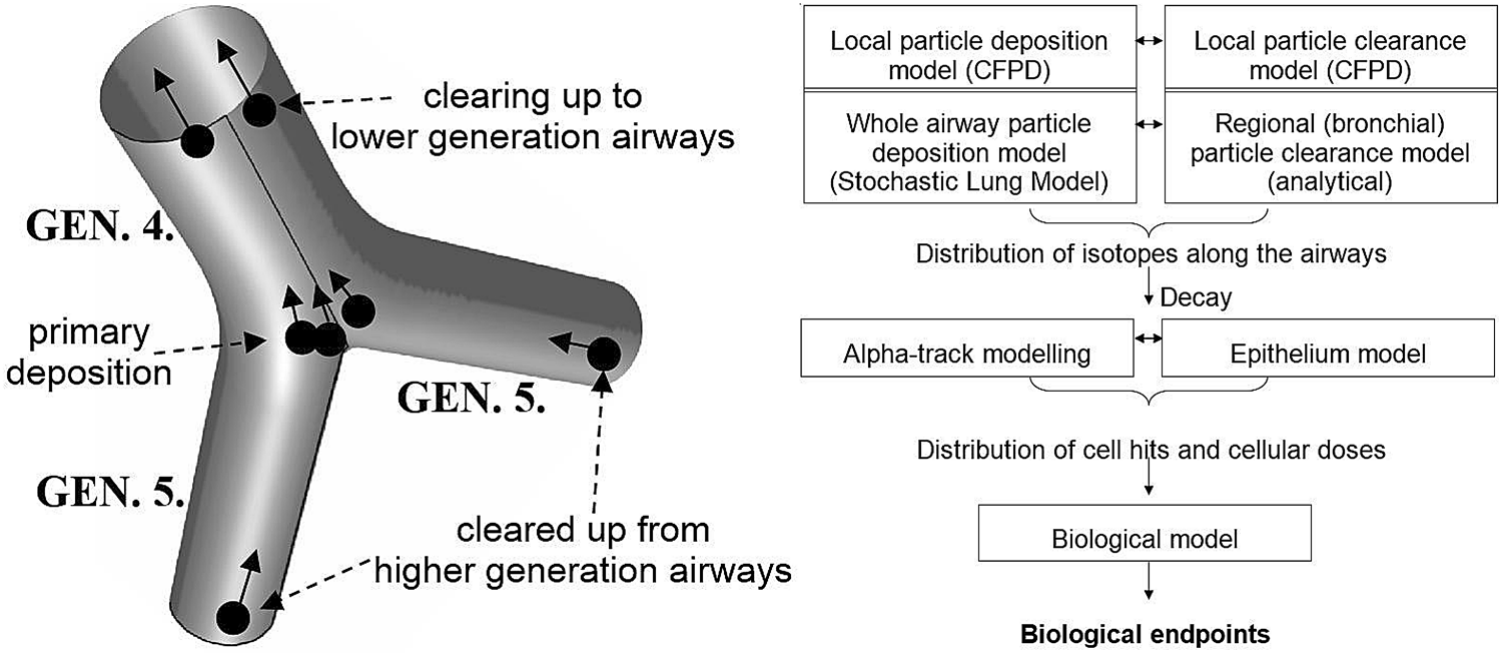
Representation of particle transport by clearance along the model airway (left) and schematic of the numerical models used (right)

As shown in the left panel of Fig. 2, particles can enter into the mucus layer directly by deposition during inhalation or exhalation, and by clearing-up from the higher generation airways (deeper airways). In the last case, the particles enter the model at the end of one of the daughter branches. At the same time, some radioactive particles can leave the bifurcation at the beginning of the parent branch clearing up to lower airway generations. Besides the airway and mucus geometries, Fig. 2 also demonstrates a scheme of the main steps of the simulations. Deposition of attached and unattached radon progenies in the whole respiratory system was modelled by the Stochastic Lung Model, as described above. Upward movement of the deposited particles (carrying radon progenies) was also modelled, to find out the fraction of particles that enter the target bifurcation per unit time (see above). Deposition in the target bifurcation was simulated in advance by CFDP techniques (see above) and the co-ordinates of the deposition sites were stored. Two thousand clearance trajectories were also calculated in advance by CFPD techniques. A particle tracking code (written in PERL programming language) was developed for the simultaneous modelling of deposition, clearance and decay in the target bifurcation geometry. The simulated particles were tracked with a time resolution of 0.1 s. At each time step, a certain number of depositing particles carrying ^218^Po, ^214^Pb and ^214^Bi isotopes were included. Their number depended on the exposure conditions (radon concentration and equilibrium factor) and their deposition efficiency. The deposition locations were randomly selected from the database of deposition co-ordinates, which were calculated in advance (see above). Radioisotopes were released at each time step from the inlets of daughter branches. Their entrance coordinates were randomly selected, while their number depended on the exposure conditions and the results of the whole lung deposition and clearance modelling (see above). Each tracked isotope could decay at each time step with a probability proportional to its activity. The location of every alpha-decay was stored. In the case of a decay, the tracked isotope changed its ID (^218^Po became ^214^Pb, ^214^Pb became ^214^Bi/^214^Po, ^214^Bi/^214^Po became ^210^Pb). Both the particles depositing in the target bifurcation and those clearing up from the deeper airways moved in the model bifurcation along one of the pre-calculated 2,000 clearance trajectories (which was the closest at the moment of deposition or entrance at the daughter branch inlet).

### Alpha-tracks and cell nucleus doses

For the modelling of the alpha energy absorbed by cell nuclei (and the related cell nucleus doses), alpha-tracks were generated and the epithelial cell nucleus structure was reconstructed. The energy absorbed by the cell nuclei was calculated from the intersection of alpha tracks with the nuclei. The starting point of the alpha-tracks were the locations of the decays, which were determined as described above. The direction of the alpha tracks was randomly selected. Near-wall (penetrating directly into the epithelial tissue) and far-wall alpha-tracks (penetrating the tissue after traversing the lumen) were distinguished. Near-wall alpha tracks resulted in an energy deposition in the mucus and tissue, while far-wall tracks caused energy deposition in the air, then in the mucus and finally in the tissue. Far-wall tracks were longer because the stopping power of alpha particles is much lower in air than in tissue. The alpha-tracks associated with the decay of ^214^Po were also longer than those assigned to ^218^Po decay, due to the higher initial kinetic energy of the alpha-particles emitted by ^214^Po. Elliptical objects represented the sensitive cell nuclei of the lung epithelium. Their size and location was derived from Mercer et al (1991, 1994). Cell nuclei of the two most radiosensitive cell types, basal and secretory cells (ICRP66), were considered. However, it should be recognized that the bronchial sections described in Mercer et al. (1991) were oblique, thus the depths of the cells in the epithelium may be overestimated. This may lead to some underestimation of doses to basal cell nuclei.

### Cell death and cell transformation modelling

To analyse the effect of the combined action of deposition and clearance in the context of cellular damage, cell death and cell transformation probabilities caused by the decaying alpha-emitting radioisotopes were calculated. These probabilities are based on the results of in vitro experiments of cell irradiations with high LET (linear energy transfer) radiation. These experiments revealed that the probability of cell survival decreases exponentially with dose to the cell nucleus. This observation can be expressed by Eq. 4

4 $$S(D) = e^{ - \gamma D} ,$$

where *S* is the cell survival probability, *D* is the dose absorbed by the cell nucleus and γ is a parameter which can be determined experimentally. In this work, the value of γ = 1.3 Gy^−1^ derived by Poncy et al (2002) on rat tracheal epithelial cells was used. Based on Eq. 4 the probability of cell death was calculated (Eq. 5).

5 $$I(D) = 1 - S(D) = 1 - e^{ - \gamma D} .$$

By the same token, based on irradiation experiments, the probability of cell transformation is proportional to the dose. In addition, a cell must survive to be able to transform, thus the transformation probability (*T*) can be described as in Eq. 6.

6 $$T(D) = \alpha De^{ - \gamma D} .$$

where α is an empirical parameter. In this work the value of α = 5 × 10^–4^ Gy^−1^ was calculated from the experiments of Miller et al. (1999) performed on CH310T1/2 cells.

It is worth noting that the experiment described in Miller et al. (1999) were performed on a mouse fibro-blast model system. In the past, this model system turned out to be quite reliable in predicting trends relevant for the estimation of health consequences in humans after radon inhalation (e.g. Brenner et al. 1995) The LET of the charged particles used in the experiment by Miller and co-workers corresponds to the LET spectrum of radon progeny alpha particles in bronchial epithelium. In Miller et al. (1999) the cell transformation probability is provided as a function of the number of alpha-hits. In the present work, the transformation probability was first plotted as a function of cellular dose and then fitted by a linear function (*αD*) to obtain the value of α.

### Radiation exposure conditions

**Fig. 3:**
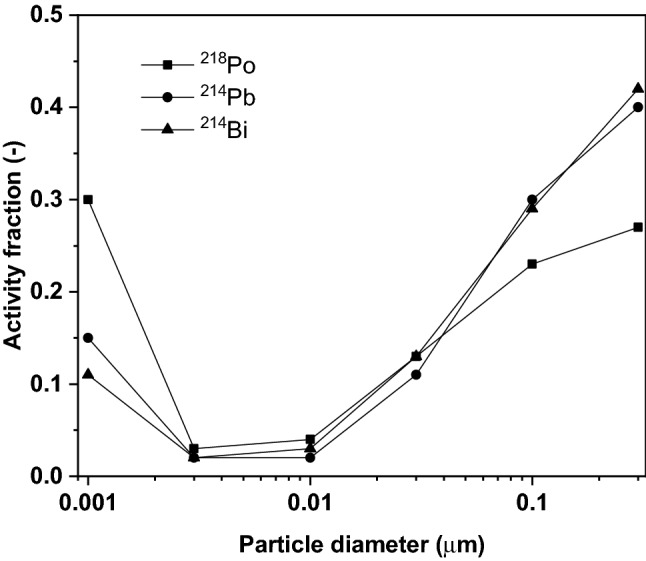
Activity size distribution of ^218^Po, ^214^Pb and ^214^Bi/^214^Po characteristic of homes, derived from data in BEIR VI (1999) publication

The numerical models described in the previous sections were applied for the case of radon progenies assuming radiation exposure conditions characteristic of homes. The main parameters of radon exposure are summarised in Table 2. This table also includes the parameters characterising the assumed breathing mode (tidal breathing of a Caucasian adult male when sitting awake) and the properties of the inhaled aerosols. The sources of the model input values are also indicated in the table. The activity-weighted size distributions of ^218^Po, ^214^Pb and ^214^Bi/^214^Po were adopted from BEIR VI (1999). The activity size distributions derived from this source can be seen in Fig. 3.

**Table 2:** Model inputs characterizing the radiation level, breathing pattern and the inhaled particles

Quantity	Value	Source or explanation
Radiation exposure		
Equilibrium radon concentration (Bq/m^3^)	46	UNSCEAR (2000)
Radioisotope concentration ratio (–)	0.58/0.44/0.29 (^218^Po:^214^Pb:^214^Bi/^214^Po)	
Equilibrium factor (–)	0.4	BEIR VI, (1999)
Breathing parameters		
Breathing mode	Nasal	ICRP 66, (1994)
Flow rate (L/min)	18	ICRP 66, (1994)
Breathing frequency (min^−1^)	12	ICRP 66, (1994)
Tidal volume (L)	0.75	ICRP 66, (1994)
Functional residual capacity (L)	3.3	ICRP 66, (1994)
Aerosol parameters		
Particle number concentration in the inhaled air (m^−3^)	7,820/51,270/25,090 (^218^Po:^214^Pb:^214^Bi/^214^Po)	Based on activity concentrations and decay half-lives
Particle diameter	See the size distribution in Fig. 3	BEIR VI

As the figure demonstrates, ^218^Po shows the highest fraction of particles around 1 nm. This is due to the lower fraction of ^218^Po attached to environmental particles, which is a consequence of the short half-life of ^218^Po. The activity size distributions were transformed into number size distributions, which were used throughout the calculations instead of the classical method of using one size for the unattached and one characteristic size for the attached progenies. The number size distribution of the particles at the entrance of the parent branch of the target bifurcation during inhalation was calculated based on the number size distribution in the surrounding air and the calculated filtration efficiency of the upper airways and the bronchial segments upstream to the selected target bifurcation. Similarly, the number size distribution of the particles entering the 5^th^ generation branches during exhalation was determined from the number size distribution in the air, the deposition fraction during inhalation and the filtration efficiency of the downstream airways during exhalation. It is worth noting that the size distribution in Fig. 3 refers to dry particles, and hygroscopic growth inside the airways may modify the size of the particles. In radon dosimetry calculations, usually it is considered that unattached radon progenies have a growth factor of 1 while attached progenies have a growth factor of 1.5–2. However, in the present study not only two particle sizes (one for unattached and one for attached) were considered, but a size distribution. Considering that below 10 nm the growth factor is close to one and the deposition fraction in the target bifurcation is very low between 10 and 500 nm and hardly changes between 100 and 500 nm, the effect of hygroscopic growth on deposition is very limited. Therefore, hygroscopicity was neglected in the present study.

### Parameters characterizing the deposition and clearance of radon progenies

The deposition and clearance of radioactive particles was characterized by different indicators. The overall deposition in a certain region/section/unit of the airways can be characterised by deposition fraction and deposition efficiency. Deposition fraction is the ratio of the number of particles deposited in a target region to the number of particles entering the whole respiratory system. By the same token, deposition efficiency is the ratio of the number of particles deposited in the target region to the number of particles entering that region. For the characterisation of the degree of inhomogeneity of the particles, particle enhancement factors can be defined. By definition, the particle enhancement factor is the ratio of the deposition density on a pre-defined surface to the average deposition density for the whole surface of the airway section considered (one bifurcation in this study). Deposition density is the number of particles on an airway surface divided by the area of that surface. To compute the distribution of local enhancement factors, the whole surface of the target bifurcations was scanned by a pre-specified surface element. Naturally, the local enhancement factor values are sensitive to the surface area of the scanning element (patch). Because in the present study the length scales of interest (alpha-track length, epithelial cell size) were of the order of tens or hundreds of micro-meters, it was plausible to choose 100 µm × 100 µm as the size of the patch (surface area = 10^–8^ m^2^).

Finally, to demonstrate the effect of the non-uniform deposition and mucociliary clearance all the calculations were performed by considering three different scenarios: (i) uniform deposition without clearance; (ii) inhomogeneous deposition without clearance and (iii) inhomogeneous deposition with clearance.

## Results and discussion

### Regional and local airway deposition of the short-lived radon progenies

**Fig. 4:**
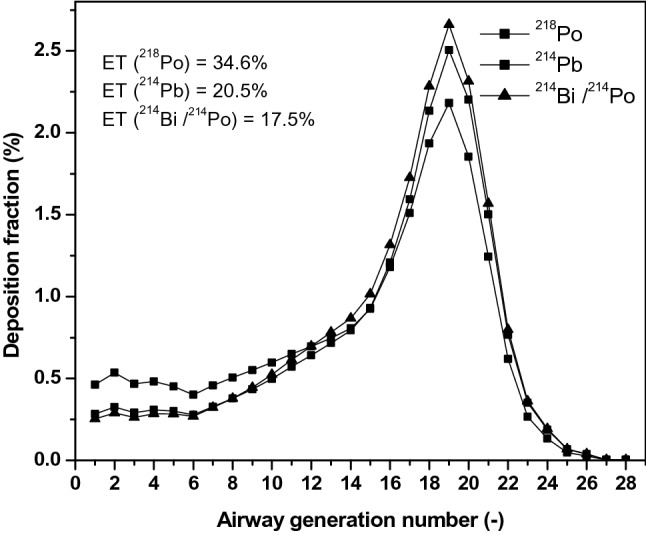
Calculated airway-generation-specific deposition fractions of ^218^Po, ^214^Pb and ^214^Bi/^214^Po. Their extrathoracic deposition fractions (ET) are also provided

As described in the Methods section, extrathoracic and airway generational deposition fractions were calculated by the Stochastic Lung Model. The results of these computations are shown in Fig. 4 separately for ^218^Po, ^214^Pb and ^214^Bi/^214^Po. It is worth noting that the deposition fraction values are provided for the whole size distribution shown in Fig. 3; thus they represent combined values of unattached and attached progenies. Taken separately, the extrathoracic deposition fraction of the unattached fraction (1 nm sized particles) would be 90%, while that of the attached fraction 5.8%. Also note that deposition fraction values of ^214^Pb and ^214^Bi are different because of different unattached fractions of these isotopes.

The deposition in the extrathoracic region is the highest in the case of ^218^Po, due to the largest fraction of this isotope in the nanometre size range. It can be calculated from these deposition fraction values that 8% of the activity represented by all the inhaled particles enter the target airway bifurcation during inhalation (through the parent branch) and 5.7% during exhalation (through the two daughter branches). These values can be used as input for the modelling of local deposition in the airway bifurcation shown in Fig. 2. The application of the CFPD deposition model using the breathing parameters in Table 2 and the specific size distribution of ^218^Po, ^214^Pb and ^214^Bi/^214^Po when these radioisotopes enter the target bifurcation resulted in a 0.8% deposition efficiency for inhalation and 0.3% for exhalation.

**Fig. 5:**
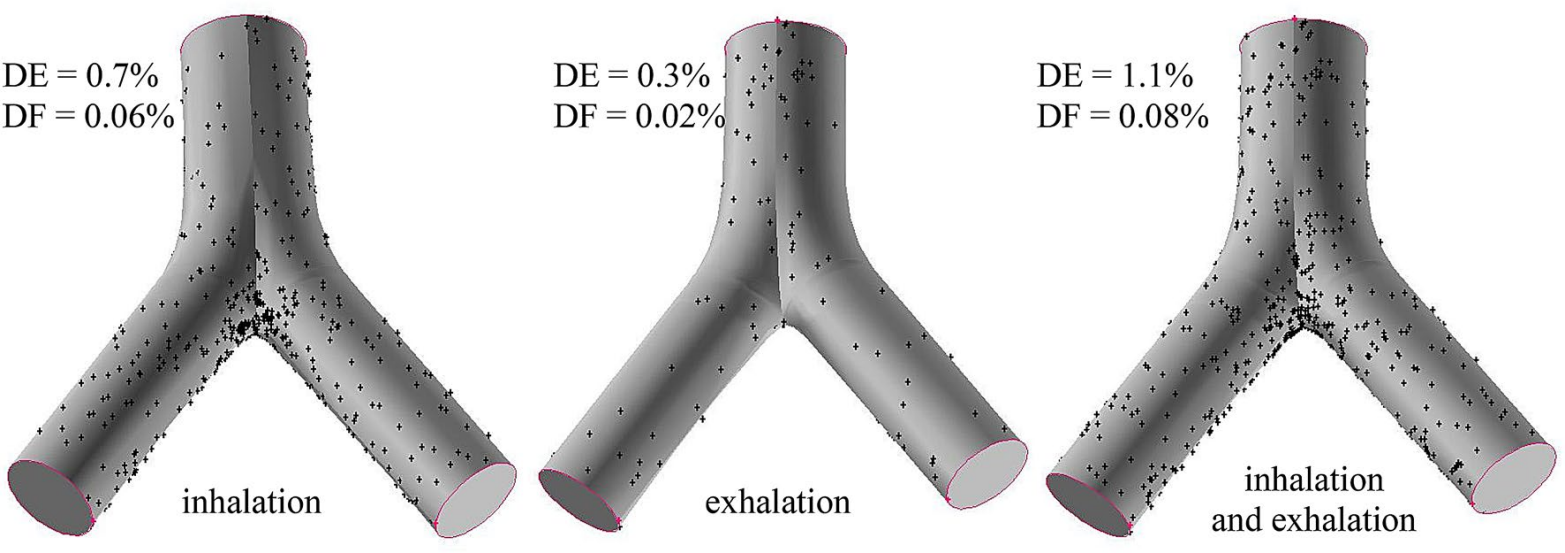
Inhalation (left), exhalation (middle) and combined (right) deposition patterns of short-lived radon progenies after 16 h of exposure in homes. *DE* deposition efficiency, *DF* deposition fraction

Figure 5 depicts an example of the computed deposition patterns for inhalation and exhalation for a breathing period of 16 h (all the three radioisotopes shown together). As the figure demonstrates, the deposition patterns are inhomogeneous (especially at) inhalation with a deposition hot spot at the peak of the bifurcation. The maximum value of the local enhancement factor is 423.1 indicating that the activity deposited over the most exposed groups of cells is two orders of magnitude higher than the average activity over the whole airway bifurcation. Combining the results of the whole airway computations (by the Stochastic Lung Model) and local deposition calculations (by CFPD techniques) shows that only 0.06% of the total inhaled activity deposits in the target bifurcation during inhalation and about 0.02% during exhalation, resulting in less than 0.1% combined (inhalation and exhalation) deposition.

### Regional and local clearance of the short-lived radon progenies

To compute the fraction of the isotopes deposited in airway generations higher than 5 and entering the model bifurcation, the regional clearance model was applied for the case of particle deposition presented in Fig. 4. The results of the simulations are presented in Table 3. In this table, the values of average airway lengths and mucus velocities are also provided. It is worth noting that a part of ^214^Pb entering the inlets of the 5th airway generation deposited originally in higher airway generations as ^214^Pb and cleared up without decaying, while another part of ^214^Pb entering the inlets of the 5th airway generation deposited originally in higher airway generations as ^218^Pb, but decayed into ^214^Pb while clearing up. By the same token, a fraction of ^214^Bi entering the inlets of the 5th airway generation deposited originally in higher airway generations as ^214^Bi and cleared up without decaying, while a second fraction of ^214^Bi entering the inlets of the 5th airway generation deposited originally in higher airway generations as ^214^Pb and decayed into ^214^Bi while clearing up. Likewise, a third fraction of ^214^Bi isotopes entering the inlets of the 5th airway generation deposited originally in higher airway generations as ^218^Pb and decayed twice (first into ^214^Pb, then into ^214^Bi) before reaching the model bifurcation.

**Table 3:** Fractions of ^218^Po, ^214^Pb and ^214^Bi nuclei originally deposited in airway generations 6–15 and cleared up to airway generation 5

Airway gen. no. of the initial deposition (–)	Average airway length (mm)	Average mucus velocity (mm/min)	Clearance time to generation 5 (s)	Fraction of ^218^Po cleared up to generation 5 (%)		Fraction of ^214^Pb cleared up to generation 5 (%)	Fraction of ^214^Bi cleared up to generation 5 (%)
6	9	0.74	730	6.29		89.39	116.23
7	7.6	0.50	1642	–		61.00	112.04
8	6.4	0.33	2806	–		36.68	88.6
9	5.4	0.22	4279	–		19.27	57.43
10	4.6	0.15	6119	–		8.61	30.16
11	3.9	0.10	8459	–		3.11	12.37
12	3.3	0.07	11,288	–		0.91	3.99
13	2.7	0.05	14,528	–		0.22	1.02
14	2.3	0.03	19,128	–		0.03	0.15
15	2	0.02	25,128	–		–	0.01
16	1.65	0.01	35,028	–		–	–

The fact that some isotopes decay during clearance while others of the same type do not decay may seem a contradiction for those who are familiar with the standard approach where constant decay rates are assumed. Obviously, average decay rates are constant; however, decay is a stochastic process. Modelling time dependence and stochasticity was possible in the present work by applying Monte Carlo techniques, which meant that while tracking the individual radioisotopes a random value from a probability distribution function wa selected. For the same time step this resulted in radioactive decay for some radioisotopes while others of the same type remained stable (e.g., ^218^Po). If the considered time period is much longer than the physical half-life of the considered radioisotope, then the probability of decay is high, but still some isotopes may not decay. In Table 3 the values exceeding 100% are a consequence of the radioactive decay law (Eqs. 1–3) and the fact that about twice as many ^214^Pb as ^214^Bi nuclei are deposited in airway generation 6. At the same time, the physical half-life of ^214^Pb is only 37% larger than that of ^214^Bi. As a result, the number of ^214^Bi nuclei will increase shortly after deposition. Naturally, after some time the number of ^214^Pb nuclei becomes so low that the number of ^214^Bi nuclei does not increase further and even starts decreasing. If the clearance time is short (as is for example the case for the clearance from airway generation 6 to airway generation 5), then the number of ^214^Bi nuclei increases, so the up-cleared fraction will be > 100% (first two values of the last column in Table 3). In contrast, if the clearance time is longer (as is for example the case for the clearance from airway generations > 7), then the up-cleared fraction will be < 100%, decreasing to zero in the case of ^214^Bi clearance from airway generation 16 to airway generation 5. Fractions of less than 0.01% were neglected, thus cleared-up ^218^Po nuclei could originate only from airway generation 6, ^214^Pb nuclei from airway generations 6–14, and ^214^Bi nuclei from airway generations 6–15.

Simulations of the local clearance pattern within the target bifurcation revealed that the mucus velocity is not uniform. While the mucus accelerated in the parent branch, there was a slow clearance zone evident at the carinal ridge of the target bifurcation, exactly where the deposition was the most intense. The inhomogeneous mucus velocity resulted in a distribution of particle residence times, defined as the time a particle spends in the target bifurcation from the entrance at one of the daughter branches to the end of the parent branch. The mean residence time turned out to be 20 min with a standard deviation of 1.7 min; however, particles entering the slow clearance zone spent up to 29 min in the target bifurcation. It is worth noting that unlike deposition, clearance is not dependent on particle diameter, thus all the radon progenies are clearing up along the same trajectories. Due to the high mucus viscosity, the particles are moving with the velocity of the mucus and their trajectories coincide with the mucus flow streamlines.

### Simultaneous deposition, clearance and decay of the short-lived radon progenies

**Fig. 6:**
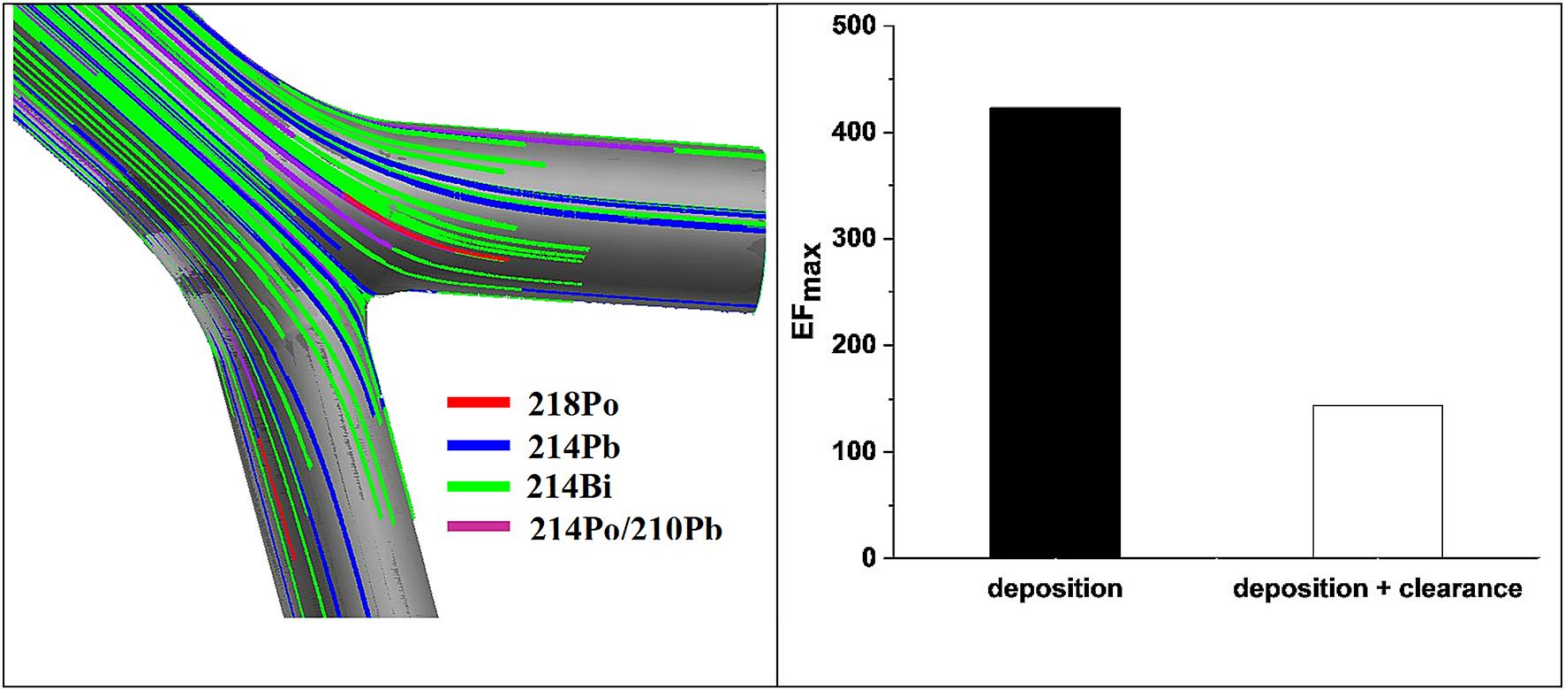
Trajectories of ^218^Po, ^214^Pb and ^214^Po inside a central airway bifurcation (left) and maximum enhancement factors assuming deposition alone or deposition and clearance

Deposition patterns shown in Fig. 5 represent cumulative values, that is, the particles are supposed immobile and inactive. In reality, however, during the exposure period a fraction of the deposited particles can leave the airway bifurcation by clearance, some particles may enter the bifurcation from the deeper airways and the activity of the radioisotopes is also changing. To account for all these phenomena, the dynamic deposition-clearance-decay model developed in the present study was applied for the exposure conditions presented in Table 2. Starting from “empty airways” (i.e., zero activity), as a result of the combined action of deposition, clearance and decay, the activity inside the target bifurcation increased gradually in the first roughly 30 min, and then oscillated around an equilibrium value. These results of the calculations can be seen in the left panel of Fig. 6. The trajectories starting at the end of the daughter branches represent nuclei originating from the deeper airways, while those starting inside the target bifurcation correspond to nuclei deposited locally (in the target bifurcation). Different colours correspond to the different nuclei. If a nucleus decayed, the colour of the trajectory changed. The location of alpha-decays and the corresponding alpha-energies (7.69 or 6 MeV) were stored. The right panel of Fig. 6 reveals that clearance leads to a decrease in the inhomogeneity of the activity distribution. The maximum value of the local enhancement factor computed solely based on deposition (423.1) decreased by a factor of about 3 due to clearance (to 143.9). This result indicates that in spite of the existence of a slow clearance area exactly at the location of deposition hot spot, mucociliary clearance decreases the radiation burden of the cells in the carinal region. Therefore, cellular doses in this region would be underestimated if uniform deposition was supposed, but overestimated, if only a realistic deposition without clearance was assumed.

### The effect of clearance on cell death and cell transformation

**Fig. 7:**
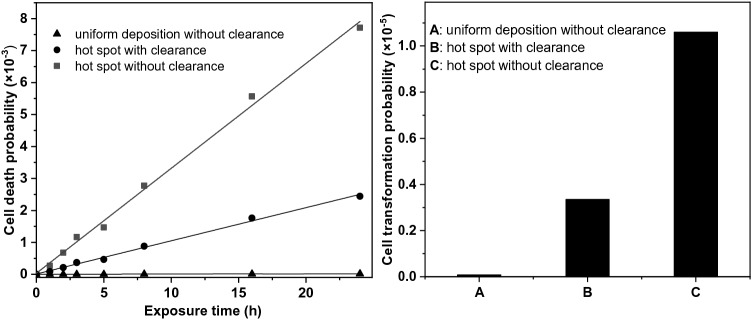
Cell death probability for different exposure times assuming uniform deposition without clearance, in the deposition hot spot neglecting clearance, and in the hot spot accounting for the effect of clearance (left); cell transformation probability assuming the same three scenarios and 24 h exposure (right)

The results of the dynamic deposition-clearance-decay model for cell death and cell transformation are depicted in Fig. 7.

The assumption of a uniform deposition (as it is currently assumed in standard dosimetry and risk models) leads to low cell death and very low cell transformation probabilities for the modelled exposure times. Considering a realistic inhomogeneous deposition did not change the average values of cell death and cell transformation much, but in the deposition hot spot the likelihood of these events was two to three orders of magnitude higher than the average values. Mucociliary clearance appears to reduce the frequency of cell death and cell transformation by factors of 2–4 highlighting the importance of modelling this effect together with deposition. The present results demonstrate that the site-specificity of deposition and inhomogeneous clearance can lead to relatively high and localized cell damage probabilities even at low levels of radiation.

The standard model including uniform deposition and clearance was not used in the present calculations because it was already published recently (Füri et al. 2020). However, comparing the results obtained with the standard model with those obtained with the present models leads to the conclusion that cell death and cell transformation probabilities for uniform deposition including clearance would fall between the cell death and transformation probabilities for uniform deposition without clearance and for the deposition hot spot with clearance (see Fig. 7).

It is worth mentioning that mucus clearance CFD simulations presented here were based on the assumption of a continuous mucus flow. The inclusion of the experimentally observed reduced clearance in a restricted zone (peak of the bifurcation) or even lack of mucus may further increase the degree of inhomogeneity of particle distribution and the related spatial heterogeneity of the biological endpoints induced by radiation exposure. Because this effect increases the degree of inhomogeneity of particle distribution and the related spatial heterogeneity of the biological endpoints, it does not compromise but further strengthen the conclusions of the present study.

It should also be noted that the dosimetric computations in this study are restricted to a single central airway bifurcation and, consequently, the results obtained may not be representative for all the airway bifurcations. As the airway generation number increases, the number of particles deposited in a single bifurcation decreases (please note that Fig. 5 sums up the deposition in all the bifurcations belonging to the same generation, thus it can be misleading from this point of view). This decrease is partly due to the decreasing airflow rate and the increasing number of airway bifurcations, but also due to the fact that fewer and fewer particles are available for deposition as one goes deeper into the airways (i.e., part of the particles have already deposited). On the other hand, clearance in the higher airway generations is less efficient, partly due to the lower mucus velocity and partly due to discontinuities of the mucus layer in the deeper airways. These are two competing effects which will cancel each other to some extent. The combined effect of deposition and clearance will depend on airway generation number, but the qualitative results obtained for generation 4–5 and the main conclusions remain unchanged.

## Conclusions

Simultaneous modelling of deposition, mucociliary clearance and radioactive decay of inhaled radon progeny allowed to simulate the airway dynamics of the short-lived radon progenies and the cellular consequences of radon progeny inhalation in a more realistic way. The accuracy of the results was improved also by considering a realistic size distribution and a time dependent approach instead of the standard method, which included only two particle sizes and a steady approach to account for clearance. The present study revealed that in the large bronchial airways not only the deposition but also the clearance patterns can be non-uniform. Clearance decreased the activity in the areas with the highest deposition density, but the activity distribution remained heterogeneously distributed. Restricted areas of the airways may receive high doses even at low macroscopic radiation levels. As a consequence, damaged cell clusters can occur at the peak of the individual airway bifurcations. Health consequences of the highly localized damage remain to be clarified.
